# A quality control method for intensity‐modulated radiation therapy planning based on generalized equivalent uniform dose

**DOI:** 10.1002/acm2.12331

**Published:** 2018-04-25

**Authors:** Haowen Pang, Xiaoyang Sun, Bo Yang, Jingbo Wu

**Affiliations:** ^1^ Department of Oncology The Affiliated Hospital of Southwest Medical University Luzhou China

**Keywords:** gEUD, IMRT, quality control, radiation planning

## Abstract

To ensure good quality intensity‐modulated radiation therapy (IMRT) planning, we proposed the use of a quality control method based on generalized equivalent uniform dose (gEUD) that predicts absorbed radiation doses in organs at risk (OAR). We conducted a retrospective analysis of patients who underwent IMRT for the treatment of cervical carcinoma, nasopharyngeal carcinoma (NPC), or non‐small cell lung cancer (NSCLC). IMRT plans were randomly divided into data acquisition and data verification groups. OAR in the data acquisition group for cervical carcinoma and NPC were further classified as sub‐organs at risk (sOAR). The normalized volume of sOAR and normalized gEUD (*a* = 1) were analyzed using multiple linear regression to establish a fitting formula. For NSCLC, the normalized intersection volume of the planning target volume (PTV) and lung, the maximum diameter of the PTV (left–right, anterior–posterior, and superior–inferior), and the normalized gEUD (*a* = 1) were analyzed using multiple linear regression to establish a fitting formula for the lung gEUD (*a* = 1). The r‐squared and *P* values indicated that the fitting formula was a good fit. In the data verification group, IMRT plans verified the accuracy of the fitting formula, and compared the gEUD (*a* = 1) for each OAR between the subjective method and the gEUD‐based method. In conclusion, the gEUD‐based method can be used effectively for quality control and can reduce the influence of subjective factors on IMRT planning optimization.

## 1 INTRODUCTION

In recent decades, intensity‐modulated radiation therapy (IMRT) has been increasingly used to enable delivery of the highest dose of radiation possible to target tumor regions while minimizing doses delivered to organs at risk (OAR), significantly improving therapeutic ratios.^1^, ^2^, ^3^, ^4^, ^5^ In IMRT plan optimization, the experience of the planning physician has a significant influence on the quality of the plan. Typically, physicians provide planners with optimization goals determined from population‐based data, Radiation Therapy Oncology Group (RTOG) guidelines, or clinical knowledge and intuition. Indeed, a lack of effective means for quality control in radiotherapy planning means that the quality of the radiotherapy depends on the experience of the radiation oncology team or center. To address this issue, retrospective optimization analysis was performed for patients who underwent IMRT for the treatment of cervical carcinoma, nasopharyngeal carcinoma (NPC), or non‐small cell lung cancer (NSCLC); the goal of this study was to propose the use of a quality control method based on generalized equivalent uniform dose (gEUD) that predicts absorbed radiation doses in organs at risk (OAR) before IMRT optimization. Several treatment planning systems have been developed that incorporate gEUD cost functions for IMRT optimization. Previous investigations have confirmed the effectiveness of gEUD cost functions for plan optimization.^6^, ^7^, ^8^ For more complex plans, more iterations are required because many parameters need to be finely tuned for dose–volume (DV)‐based objective functions. gEUD was developed with fewer parameter settings to improve the quality of plans.^9^ The phenomenological form of gEUD is as follows:

$$\text{gEUD}={\left(\frac{1}{m}\sum_{i=1}^{m}{d_{i}}^{a}\right)}^{\frac{1}{a}}$$

where, m is the number of voxels in the anatomical structure of interest, d_i_ is the dose in the ith voxel, and a is the tumor or normal tissue specific parameter. For *a* = ∞, gEUD is equal to the maximum dose; for *a* = −∞, gEUD is equal to the minimum dose; for *a* = 1, gEUD is equal to the arithmetic mean dose; and for *a* = 0, gEUD is equal to the geometric mean dose. Because the mean dose is a critical evaluation condition for OAR, gEUD (*a* = 1) was used in this study as a restrictive and evaluative condition for OAR to evaluate the quality of radiotherapy planning and reduce the influence of subjective factors on the quality of the radiotherapy plan.

## 2 MATERIALS AND METHODS

Previous studies have shown a correlation between the radiation dose absorbed by the OAR and the spatial position of the target region.^10^, ^11^ In this study, the prescription dose of the target region was considered to be the dose of the intersection area of the OAR and target area. The OAR included the bladder, rectum, and femoral head in patients with cervical carcinoma; the inner ears, oral cavity, parotid gland, larynx, postcricoid region of the hypopharynx, and esophagus in patients with NPC; and the lung in patients with NSCLC.

### 2.A Patient information

Patients with cervical cancer, NPC or NSCLC who underwent IMRT planning at our department were randomly selected and divided into data acquisition and data verification groups. The data acquisition group included 50, 65, and 50 patients who underwent IMRT for the treatment of cervical carcinoma, NPC, and NSCLC, respectively. The data verification group included 20 patients with each disease.

### 2.B Target delineation

Target delineation was performed in accordance with International Commission on ICRU Reports 50 and 62.^12^, ^13^ For cervical carcinoma, the clinical target volume (CTV) was delineated by a radiation oncologist. The margin of CTV to the radiotherapy planning target volume (PTV) was 1 cm in the superior–inferior direction and 0.8 cm in other directions.

For NPC, a radiation oncologist delineated the gross tumors volume (GTV) and clinical target volumes 1 and 2 (CTV1 and CTV2). The GTVs included visible tumors and/or enlarged or suspicious lymph nodes, CTV1 included the high‐risk regions surrounding the primary tumors and the upper neck nodes at risk, and CTV2 included the mid‐lower nodes at risk. The margins of the GTVs, CTV1, and CTV2 were all extended outwards by 3 mm to generate the PGTVs, PCTV1, and PCTV2.

For NSCLC, the GTV and CTV were delineated by a radiotherapist, where PTV = CTV + ITV (internal target volume) + 6 mm. The same radiation oncologist performed target delineation for all patients.

### 2.C Prescription dose and plan evaluation

For cervical carcinoma, the prescription dose for the PTV was 48.0–50.4 Gy, and the per fraction doses was 1.80 Gy, which was selected in accordance with the RTOG 0418.^14^

For NPC, the prescription dose was in accordance with the regulations of the RTOG 0615 and RTOG 0225.^15^, ^16^ The prescription dose for the PGTVs was 70 Gy, and the per fraction doses was 2.12 Gy. The PCTV1 treatment dosage was 60–66 Gy, while the per fraction doses was 1.80–2.00 Gy. The PCTV2 prescription dose was 54–56 Gy, and per fraction doses was 1.64–1.70 Gy.

For NSCLC, the prescription dose was selected in accordance with the regulations of the RTOG 0617.^17^ The prescription dose for the PTV was 60.0–70.0 Gy, and the per fraction doses was 2.00 Gy.

To evaluate the dose distribution of the target, the following parameters were calculated for the PTV: minimal dose delivered to 98% of the target volume (D98%), maximum dose delivered to 2% of the target volume (D2%), conformation number (CN), and homogeneity index (HI) according to ICRU report 83.^18^ The CN was defined using the following equation:^19^

$$\text{CN}=\frac{TV_{\mathrm{RI}}}{\mathrm{TV}}\times \frac{TV_{\mathrm{RI}}}{V_{\mathrm{RI}}}$$

where CN is the conformation number, TV_RI_ is the target volume receiving the reference isodose, TV is the target volume, and V_RI_ is the volume of the reference isodose. The CN ranged from 0 to 1, where 1 was the ideal value. The HI was calculated using the following equation:^18^

$$\text{HI}=\frac{D2\%-D98\%}{D50\%}$$

where D2% is the near‐maximum dose, D98% is the near‐minimum dose, and D50% is the dose received by half of the PTV. An HI of 0 indicated that the absorbed dose distribution was almost homogenous.

### 2.D Limiting requirements for OAR

For cervical carcinoma, the bladder and rectum were restricted to V45 ≤ 50%, V50 ≤ 50% for individual patients, and V50 of the femoral head ≤5%, thus controlling the volume of “hot spots”.^14^ A window width of 300–500 HU and a window level of 30–50 HU is recommended when the bladder and rectum are delineated, a CT scan slice thickness of 5 mm is also recommended.

For NPC, the mean dose to a unilateral inner ear was ≤45.0 Gy, the mean dose to the oral cavity was ≤40.0 Gy, and the mean dose to the parotid gland was ≤26.0 Gy. In cases where the intersection volume of the parotid gland and PCTV2 was too large, the unilateral D50% was kept at <30.0 Gy or as low as possible, the mean dose to the larynx was ≤45.0 Gy, the mean dose to the postcricoid region of the hypopharynx at ≤45.0 Gy, and the mean dose to the esophagus at ≤45.0 Gy.^15^, ^20^, ^21^, ^22^ A window width of 1500–2000 HU and window level of 400–450 HU is recommended when the inner ear is delineated. A window width 300–350 HU and window level of 30–50 HU is recommended when the other OAR are delineated; the recommended CT scan slice thickness is 2.5 mm.

For NSCLC, the V20 and V5 of the lung were kept below 30% and 65%, respectively. The mean dose was no more than 20 Gy.^23^ A window width of 1300–1700 HU and window level of −600 to −800 HU is recommended when the lung is being delineate, and the CT scan slice thickness is recommended to be 2.5 mm.

### 2.E IMRT plan design for the data acquisition group

We selected the 6 MV x‐ray co‐planar 7‐beam average divisions (gantry angle at 180°, 128°, 76°, 332°, 280°, and 228°) for cervical carcinoma, 6 MV x‐ray co‐planar 9‐beam average divisions (gantry angle at 160°, 120°, 80°, 40°, 0°, 320°, 280°, and 240°) for NPC, and the 6 MV x‐ray co‐planar 5~8‐beam (gantry angles depending on the tumor location) for NSCLC via the Direct Machine Parameter Optimization (DMPO) algorithm and dose engine (the grid resolution of a 4‐mm dose grid with a 2‐mm fluence grid was used, including the heterogeneity correction) on the Pinnacle^3^ 8.0‐m (Philips, Fitchburg, WI) treatment planning software.

For cervical carcinoma the PTV was extended externally to several rings (0.5 cm in width, considering the large volume of OARs) prior to plan optimization. For NPC the PCTV2 was extended externally to several rings (0.3 cm in width, considering the small volume of OARs) prior to plan optimization.

**Figure 1:**
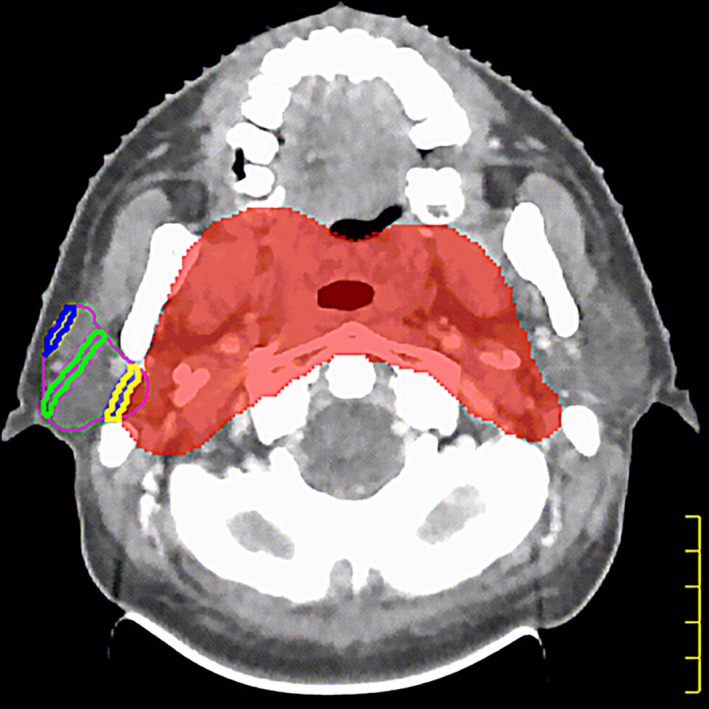
The red shadow indicates PCTV2, the purple line indicates the right parotid gland, the yellow line indicates the intersection of ring1 and the right parotid gland (the nearest ring area to the right parotid gland), the green line indicates the intersection of ring4 and the right parotid gland (the middle of ring area to the right parotid gland), and the blue line indicates the intersection of ring7 and the right parotid gland (the farthest ring area to the right parotid gland). There are eight rings in total.

The intersection areas of ring1~ringn and OAR (ring1~ringn∩OAR) were considered to be independent sub‐organs at risk (sOAR); gEUD (*a* = 1) for sOAR was regarded as the optimization restrictive condition to make constant adjustments for values and weight. The dose of the intersection area of OAR and the target area was recognized as the prescription dose, and the dose of the target area was guaranteed to keep the absorbed dose of each OAR as low as possible so that the requirements of the evaluation were met. When numerous sOAR were present, sOAR weight parameters were adjusted to smaller values (with respect to the target region). Figure 1 illustrates the intersection area of ring1∼ringn and the right parotid gland in a patient with NPC (case 39).

Given the distinctiveness of the lung's position for NSCLC treatment, this study did not divide the OAR into sOAR in patients with NSCLC. During optimization, the values and weights of V5 and V20 were adjusted repeatedly. Under the precondition of meeting the target area's evaluation conditions, V5, V20, and the mean dose to the lung were minimized as much as possible.

### 2.F Fitting formula

The following methods were used for cervical carcinoma and NPC in the data acquisition group. Using SPSS 13 software, the Spearman rank correlation test was used to analyze the correlation between volume of sOAR and gEUD (*a* = 1) of each OAR, and a multiple linear regression analysis was used to fit the experimental data for the normalized volume of sOAR (V_ring1∼ringn∩OAR_/V_OAR_) and normalized gEUD (gEUD/D_prescription_, *a* = 1, for cervical carcinoma, D_prescription_ is the prescription dose of PTV; for NPC, D_prescription_ is the prescription dose of PCTV2) of each OAR. The following formula was obtained:

(1) $$Y=H_{c}+H_{0}\times V_{0}+H_{1}\times V_{1}+\cdots +H_{n}\times V_{n}$$

where Y is the normalized gEUD (gEUD/D_prescription_) of OAR when *a* = 1; V_0_ for cervical carcinoma patients is the normalized intersection volume of the PTV and OAR (V_PTV∩OAR_/V_PTV_); V_0_ for NPC patients is the normalized intersection volume of the PCTV2 and OAR (V_PCTV2∩OAR_/V_PCTV2_) of each patient; V_1_ is the normalized intersection volume of ring1 and OAR (sOAR_1_) of each patient; and analogously, V_n_ is the normalized intersection volume of ringn and OAR (sOAR_n_) of each patient; H_c_ and H_0_–H_n_ are fitting parameters.

For NSCLC, using SPSS 13 software, the Spearman rank correlation test was used to analyze the correlation between the intersection volume of the PTV and lung, the maximum diameter of the PTV (left–right, anterior–posterior, and superior–inferior), and gEUD (*a* = 1) of the lung, In addition multiple linear regression analyses were used to fit the experimental data for the normalized intersection volume of the PTV and lung, the maximum diameter of the PTV (left–right, anterior–posterior, and superior–inferior), and normalized gEUD (gEUD/D_prescription_) of the lung (*a* = 1). The following formula was obtained:

(2) $$Y=B_{c}+B_{0}\times V_{0}+B_{1}\times D_{1}+B_{2}\times D_{2}+B_{3}\times D_{3}$$

where Y is the normalized gEUD (gEUD/D_prescription_, *a* = 1) of the lung, V_0_ is the normalized intersection volume of the PTV and lung (V_PTV ∩lung_/V_PTV_), D_1_ (cm) is the maximum diameter of the PTV (left–right), D_2_ (cm) is the maximum diameter of the PTV (anterior–posterior), and D_3_ (cm) is the maximum diameter of the PTV (superior–inferior). B_c_, B_0_, B_1_, B_2_, and B_3_ are fitting parameters.

### 2.G IMRT Plan design for the data verification group

Optimization was carried out on 20 patients with each disease using two methods: a subjective method (Method 1) and a gEUD‐based optimization method (Method 2). In Method 1, conventional, experience‐based limits (optimization goals referenced from population‐based data, RTOG guidelines, or clinical knowledge and intuition) were used for the investigated OAR. Method 2 was used to gEUD (*a* = 1) as the optimization parameter for each OAR, the planner had to repeatedly adjust the optimized parameters of the OARs and target area (gEUD for each OAR was calculated using Formula 1 or 2 just as the reference optimization parameter). The dose of the target area and OARs were guaranteed to satisfy the requirements of the evaluation in Method 1 and Method 2. Using SPSS 13 software, statistical differences were determined using a two‐sided paired t‐test. Differences with a p value of <0.05 were considered significant.

## 3 RESULTS

The volume of the sOAR had a significant correlation with gEUD (*a* = 1) of each OAR for cervical carcinoma and NPC in the data acquisition group (all p < 0.05). For Formula 1, H_c_ and H_0_–H_n_ values for different OAR are presented in Table 1. Table 2 summarizes the r‐squared of different OAR for IMRT after the data were fitted using Formula 1. For the bladder, the *P*‐value was 0.021; for the remaining OAR, *P*‐values were <0.0001. Taken together, the *r*‐squared and *P*‐values suggested that Formula 1 was a good fit.

**Table 1:** H_c_ and H_0_‐H_n_ values for IMRT

H	OAR								
	Bladder	Rectum	Femoral head	Inner ear	Oral cavity	Parotid	Larynx	Hypopharynx	Esophagus
H_c_	−0.183	−0.290	−0.293	0.427	0.521	0.432	0.549	0.728	0.614
H_0_	1.176	1.396	0.297	0.714	0.605	0.842	−0.631	0.386	0.582
H_1_	1.513	1.357	1.328	0.321	0.558	0.457	0.633	0.231	0.194
H_2_	1.552	1.358	1.329	0.725	0.523	−0.081	0.561	0.263	0.322
H_3_	1.543	0.878	1.739	−0.167	−0.512	0.175	0.165	−0.691	−0.056
H_4_	0.875	−0.060	0.004	0.197	0.329	0.156	−0.224	0.000	−0.044
H_5_	−1.496	2.871	1.046	−0.191	0.609	0.500	0.295	−0.544	−0.313
H_6_	−0.689	−4.775	0.052	0.226	0.312	−1.275	−0.543	−0.306	−0.053
H_7_	8.645	–	2.094	−0.864	−0.495	0.472	0.400	−0.395	−0.982
H_8_	−5.048	–	0.395	–	−2.100	−0.381	−1.032	–	0.098
H_9_	6.610	–	0.352	–	−0.124	−0.043	0.421	–	–
H_10_	−8.565	–	−0.472	–	2.991	−0.586	–	–	–
H_11_	8.438	–	–	–	−1.259	–	–	–	–
H_12_	−3.646	–	–	–	0.202	–	–	–	–
H_13_	–	–	–	–	−2.686	–	–	–	–
H_14_	–	–	–	–	0.483	–	–	–	–
H_15_	–	–	–	–	−1.096	–	–	–	–

**Table 2:** The *r*‐squared for OAR

*r*‐squared	OAR								
	Bladder	Rectum	Femoral head	Inner ear	Oral cavity	Parotid	Larynx	Hypopharynx	Esophagus
*r*‐squared	0.939	0.941	0.943	0.968	0.916	0.954	0.845	0.912	0.956

The intersection volume of the PTV and lung or the maximum diameter of the PTV (left–right, anterior–posterior, and superior–inferior) significantly correlated with gEUD (*a* = 1) of lung for NSCLC in the data acquisition group (all *P* < 0.05). For Formula 2, *B*_c_ = −0.01, *B*_0_ = 0.189, *B*_1_ = 0.008, *B*_2_ = 0.002, and *B*_3_ = 0.012. The r‐squared was 0.904 (*P* < 0.0001) suggested that Formula 2 was a good fit.

For the data verification group, gEUD_plan1_ was calculated using Method 1, gEUD_plan2_ was calculated using Method 2, and gEUD_pred_ was calculated using Formula 1 or 2. Using δ_1_ = (gEUD_plan1_−gEUD_pred_), δ_2_ = (gEUD_plan2_−gEUD_pred_), the mean δ_1_ and δ_2_ values for each OAR in the present study are shown in Table 3. The mean δ_1_ values of each OAR were significantly higher than the mean δ_2_ values (*P* < 0.05); the mean gEUD_plan1_ values and gEUD_pred_ values of each OAR were significantly different (*P* < 0.05); the mean gEUD_plan2_ values and mean gEUD_pred_ values of each OAR were not significantly different (*P* > 0.05). For cervical carcinoma, NPC, and NSCLC IMRT plans, no significantly different were observed in the D98%, CN, and HI of the target region obtained using Methods 1 and 2 (*P* > 0.05). The CN and HI for each PTV in the NPC are shown in Table 4.

**Table 3:** The mean δ values (cGy)

δ	OAR									
	Bladder	Rectum	Femoral head	Inner ear	Oral cavity	Parotid	Larynx	Hypopharynx	Esophagus	Lung
δ_1_	364 ± 150	464 ± 284	554 ± 206	325 ± 209	211 ± 291	217 ± 213	729 ± 201	580 ± 249	592 ± 257	318 ± 247
δ_2_	137 ± 70	168 ± 62	158 ± 101	57 ± 79	100 ± 156	98 ± 127	39 ± 104	81 ± 124	190 ± 118	148 ± 128

**Table 4:** Conformation number (CN) and homogeneity index (HI) of nasopharyngeal carcinoma in the data acquisition group for each PTV

	Method1	Method2	*P*
CN			
PGTVs	0.58 ± 0.09	0.60 ± 0.08	0.65
PCTV1	0.63 ± 0.08	0.64 ± 0.07	0.57
PCTV2	0.66 ± 0.07	0.67 ± 0.06	0.66
HI			
PGTVs	0.06 ± 0.03	0.07 ± 0.03	0.89
PCTV1	0.15 ± 0.04	0.16 ± 0.04	0.92
PCTV2	0.12 ± 0.03	0.14 ± 0.02	0.62

## 4 DISCUSSION

Quality control of the planned dose of radiation absorbed by OAR during radiotherapy is critically important.^24^, ^25^, ^26^, ^27^, ^28^, ^29^ In the present study, OAR for cervical carcinoma and NPC were divided into sOAR and a multiple linear regression analyses were used to fit the experimental data for sOAR volumes and gEUD (*a* = 1). For NSCLC, a multiple linear regression analyses were used to fit the experimental data for the intersection volume of the PTV and lung, the maximum diameter of the PTV (left–right, anterior–posterior, and superior–inferior), and gEUD (*a* = 1). The *r*‐squared and *P* values indicated that the fitting formula was a good fit. The volume of sOAR had a significant correlation with the gEUD (*a* = 1) of each OAR for cervical carcinoma and NPC (all *P* < 0.05). In addition, the intersection volume of the PTV and lung or the maximum diameter of the PTV (left–right, anterior–posterior, and superior–inferior) had a significant correlation with the gEUD (*a* = 1) of lung for NSCLC (all *P* < 0.05) in the data acquisition group which indicated that using sOAR volumes or diameters are able to predict gEUD (*a* = 1). Our method is a relatively simple mathematical model, which does not require buying new modules of treatment planning software (TPS) or extracting the distance of each sampling point of the OAR with the dose information.

The mean gEUD_plan2_ values and mean gEUD_pred_ values of each OAR were not significantly different (*P* > 0.05) which indicated that both Formula 1 and 2 were able to accurately predict achievable gEUD (*a* = 1). Since IMRT was used and beam apertures are discrete around the patient, the result might be different depending on the volume of the bladder covered, and considering that the beams cover a large volume. This could explain the larger *P*‐value (*P* = 0.021); however as *P* < 0.05, it indicates that Formula 1 is still valid. The mean δ_1_ values of each OAR were significantly higher than the mean δ_2_ values (*P* < 0.05), while no statistically significant differences were observed in the D98%, CN, and HI of the target region using Methods 1 and 2, This shows that Methods 2 can achieve the same target region quality, and better quality for each OAR compared to Method 1.

The process model for the acquisition of sOAR volumes can be edited into scripts to improve efficiency. Simultaneously, more sample data can be added to the original data to yield an updated and more accurate fitting formula. The methods used in the present study can also be applied before planning in other disease states to obtain the predicted value of gEUD when *a* = 1, that is, as a standard for quality control in radiotherapy planning.

Consider δ, δ closer to 0 indicated a closer relationship between the planned and predicted values of gEUD (*a* = 1). The δ threshold can be set for standardizing the gEUD (*a* = 1) of each OAR. The δ above the threshold needs to be further optimized until a satisfactory δ is obtained under the conditions of the target prescription evaluation.

Importantly, there are many other factors that affect the quality of radiotherapy planning, and these factors should be considered in future efforts toward IMRT plan optimization. For example, to better protect OAR, dose reduction around the target area should be discussed by physicists and doctors to reach an appropriate conclusion for individual cases.

Further research through multicentered studies with larger data sets collected from different planning systems and OAR in different diseases is required. If the data set is large enough, the neural network fitting method can be employed to provide a more accurate fit.

## 5 CONCLUSION

gEUD can be predicted on the basis of the volume of sOAR, the intersection volume of the PTV and OAR, or the maximum diameter of the PTV (left–right, anterior–posterior, and superior–inferior). The gEUD‐based method can be used for quality control means and reduce the influence of subjective factors on IMRT planning optimization.

## CONFLICTS OF INTEREST

The authors declare no conflict of interest.
